# Stunned Myocardium as a Sequela of Acute Severe Anemia: An Adult Simulation Case for Anesthesiology Residents

**DOI:** 10.15766/mep_2374-8265.11432

**Published:** 2024-09-06

**Authors:** David Ryusuke Okano, Bryan Ko, Marelle Giuliano, Sally Mitchell, Johnny Cartwright, Christopher Moore, Tanna Boyer

**Affiliations:** 1 Associate Professor, Department of Anesthesia, Tokyo Women's Medical University; 2 Fourth-Year Medical Student, Indiana University School of Medicine; 3 Second-Year Resident, Department of Anesthesia, Indiana University School of Medicine; 4 Associate Professor, Department of Anesthesia, Indiana University School of Medicine; 5 Associate Director of Simulation, Department of Anesthesia, Indiana University School of Medicine; 6 Simulation Technician, Department of Anesthesia, Indiana University School of Medicine; † Co-primary authors

**Keywords:** Crisis Resource Management, Stunned Myocardium, Surgical Blood Loss, Transfusion, Anesthesiology, Clinical Skills Assessment/OSCEs, Clinical Teaching/Bedside Teaching, Simulation

## Abstract

**Introduction:**

Anesthesiologists develop anesthetic plans according to the surgical procedure, patient's medical history, and physical exams. Patients with ischemic heart disease are predisposed to intraoperative cardiac complications from surgical blood loss. Unanticipated events can lead to intraoperative complications despite careful anesthesia planning.

**Methods:**

This anesthetic management simulation was developed for the anesthesiology residency curriculum during the first clinical anesthesia year (CA 1/PGY 2 residents). A total of 23 CA 1 residents participated. A 50-minute encounter focused on a 73-year-old male who presents for an elective total hip replacement and develops acute myocardial stunning in the setting of critical acute blood loss and a delay in the transportation of blood products.

**Results:**

One hundred percent of the residents felt the simulation was educationally valuable in the immediate postsimulation survey (Kirkpatrick level 1). The follow-up survey showed that 100% of residents felt the simulation increased their knowledge of managing acute cardiac ischemia (Kirkpatrick level 2), and 93% felt it increased awareness and confidence in similar real-life situations that positively affected patient outcomes (Kirkpatrick level 3).

**Discussion:**

Our simulation provides a psychologically safe environment for anesthesiology residents to develop management skills for acute critical anemia and cardiogenic shock and foster communication skills with a surgery team.

## Educational Objectives

By the end of this activity, learners will be able to:

1. Perform an efficient patient encounter in the preoperative area, focusing on the implications of coronary artery disease and anti-platelet therapy for coronary stents.
2. Establish an anesthesia plan appropriate for the proposed surgery and the presented patient.
3. Recognize the signs and symptoms of acute surgical blood loss and treat accordingly.
4. Demonstrate effective communication with surgeons and the surgical team in the OR.
5. Adapt to an unexpected delay in blood product delivery and manage unstable hemodynamics.
6. Differentiate between hypovolemic shock versus anemic shock versus cardiogenic shock.
7. Manage intraoperative cardiogenic shock and subsequent ischemic cardiac events due to untreated anemia.
8. Demonstrate effective communication with the cardiologist.

## Introduction

Patients with ischemic heart disease are predisposed to intraoperative cardiac complications from surgical blood loss. Anesthesiologists evaluate patients preoperatively to anticipate and identify potential cardiac risks depending on the type of surgical procedure, the patient's tolerance to anemia, and subsequent ischemia to vital organs and develop the anesthetic plan to minimize intraoperative complications ^1–6^

We introduce an anesthetic management scenario focused on acute myocardial ischemia in critical acute blood loss. A delay in blood product transportation creates unanticipated long waits, and prolonged silent ischemia results in myocardial stunning, leading to adverse clinical outcomes.^3,7^

Myocardial stunning is a condition where the ventricles suffer a period of prolonged dysfunction after a brief interval of nonlethal ischemia. The myocardium eventually fully recovers but may require inotropic or mechanical support during the interim.^1,2,8,9^ Echocardiogram can assist with diagnosis, but in an emergent OR setting, diagnosis relies more on vital sign trends and identification of cardiac stressors.^10^ We chose myocardial stunning over infarction because it is more nuanced and challenging for early anesthesia trainees to detect.

Treatment goals of acute cardiogenic shock center around protecting the heart and improving blood flow to essential organ systems. Given the various circumstances where cardiogenic shock presents, standardized treatment algorithms have been challenging to produce.^8,9^ Initial treatment includes addressing any known causes of the shock state, such as fluid and blood product resuscitation in the setting of acute blood loss. Inotropic, chronotropic, and vasopressor drugs may be used to maintain cardiac output and blood pressure. Beta-blockers may be required to prevent tachycardia to maintain coronary perfusion. In the absence of formal guidelines for cardiac shock treatment, current literature suggests that a goal mean arterial pressure of 65–100 mmHg should be maintained.^8^

This scenario is designed to educate anesthesiology residents on how to evaluate risk factors for cardiac complications and develop a plan to mitigate those risks. Residents learn how to detect signs of intraoperative acute blood loss, differentiate anemic/hypovolemic shock from cardiogenic shock, communicate effectively with the surgical team, and adapt the anesthetic plan to manage emergent changes in patient status.

Peer-reviewed sources regarding managing acute critical anemia without access to blood products are limited. There are multiple educational resources within *MedEdPORTAL* addressing myocardial infarction, including those on how to teach chest pain.^11,12^ Other educational resources within *MedEdPORTAL* teach about acute ST elevation myocardial infarctions,^13,14^ but only one is set within the OR.^15^ *MedEdPORTAL* searches of *stunned myocardium* and *acute blood loss* produce zero relevant results across all specialties. A broader search outside *MedEdPORTAL* produced zero educational materials on the topic of stunned myocardium, indicating that this simulation fills a need within the medical training community.

## Methods

### Development

The anesthesiology residency program at the Indiana University School of Medicine has 23–26 trainees per class. This scenario was introduced in 2013 and has run every successive year for the target clinical anesthesia year 1 (CA 1) residents. Previous iterations of the simulation used informal written assessment that lacked in response rate. After 10 years of repeated updates and positive informal feedback from residents, we created a formal assessment for publication that would allow us to introduce this scenario to other simulation facilities. All data below were gathered from one iteration of this curriculum during the 2022–2023 academic year. Details of the scenario are available in Appendix A.

### Equipment/Environment

• Two patient rooms for preoperative and OR setting
• Simulator mannequin supine on the operating table
• Simulated vital sign monitor with an interconnected software interface
• Fully functioning anesthesia machine
• Orthopedic prop surgical kit on a mayo stand
• Suction and simulated blood
• Simulated bags of packed red blood cells
• Adult code cart
• Anesthesia cart (including a stethoscope, airway devices, a standard set of simulated anesthesia medications, and assorted syringes and needles)

### Personnel

The minimum requirements for this scenario were one anesthesiologist (target learner), one patient standardized participant (SP), and one surgeon SP. To increase realism, additional SP roles could be added, such as resident surgeons, scrub nurses, circulator nurses, and so on. The mannequin and monitors were programmed and operated by two simulation operation specialists. The faculty facilitator was also in the control room during the simulation and gave verbal cues and instructions through headsets worn by the SPs.

Given the limited budget in our simulation curriculum, we opted to use volunteer SPs, who were the non-target learner CA 1 anesthesia trainees. We realize this is not the gold standard in simulation education (which would be using paid standardized actors as SPs). Still, we are happy to offer this as an alternative for programs whose simulation departments may also lack funding and resources.

### Implementation

First, the patient SP was briefed on their role (Appendix B) and waited in the preoperative area. The learner anesthesiologist was briefed (Appendix C) and given orientation instructions for the simulated OR room, anesthesia equipment, and medications for the proposed surgery. The surgeon SP was briefed (Appendix D) and waited outside the preoperative area. The learner was then instructed to evaluate and consent the patient in the preoperative area where the learner had been briefed on physical exam and baseline laboratory findings (Appendix B).

The learner anesthesiologist and surgeon then entered the OR. To anticipate future scenario progression, the instructor asked the learner anesthesiologist to verbalize their thought process and next steps. To administer medications, the learner anesthesiologist verbalized the drugs and dosages. Drug administration could also be simulated by injecting saline into the mannequin's IV line using labeled syringes. Appendices E and F were given to the learner if the relevant test results were requested.

### Debriefing

The facilitator reviewed each learner's completion of the critical actions checklist (Appendix G). Open-ended questions were posed to the learners regarding their assessment of the scenario and performance to encourage active discussion from all participants and elicit reflection via debriefing with good intentions. Facilitators and learners discussed best practices regarding the implications of coronary artery disease, cardiogenic versus hypovolemic shock, and acute critical anemia.

The following elements and talking points were addressed during the debrief with the learners. Detailed discussion points are presented in Appendix H.

1. Evaluation of the patient in the preoperative area
2. Differentiating hypovolemia versus anemia versus cardiogenic shock
3. Adaptation to the delay in receiving blood products
4. Management of cardiogenic shock under critical anemia complicated with delay in blood transfusion
5. Communication with the surgical team

### Assessment

Formative feedback was given to the learners during debriefing. Current CA 1 residents in this curriculum were asked to provide anonymous, immediate postsimulation evaluation (survey A). Four months later, CA 1 residents were invited to respond to anonymous follow-up survey B to assess the efficacy of the simulation in long-term behavior changes and knowledge retention. Survey B was simultaneously distributed to CA 2 and CA 3 residents who had experienced the same simulation when they were CA 1s. Both A and B survey links were distributed via institutional email. Since this project involved anesthesia residents, we obtained IRB exemption (Indiana University Office of Research Compliance Committee, 17860, January 11, 2023).

## Results

The response rate of CA 1 residents (*n* = 23) to survey A was 87% (20 of 23; Table 1). Survey items were rated on a 5-point Likert scale (1 = *strongly agree,* 2 = s*omewhat agree,* 3 = *neither agree nor disagree,* 4 = *somewhat disagree,* 5 = *strongly disagree*). The mean and standard deviation values were calculated using raw Likert scores. Overall results of survey A were positive, with most participants indicating that the simulation was valuable to their education and improved their understanding of myocardial stunning in the acute blood loss anemia setting.

**Table 1.**
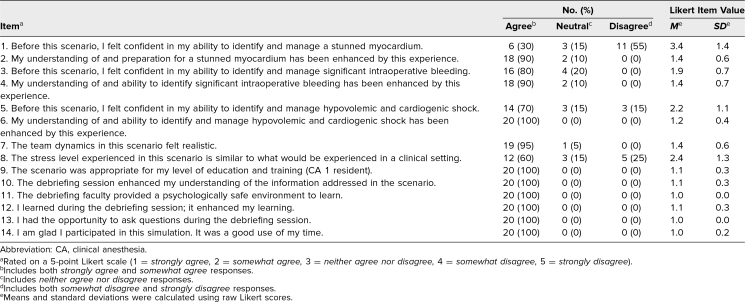
Distribution of Responses to Survey A, Mean Scores, and Standard Deviations (*N* = 20)

The total response rate to survey B was 65% (44 of 68), with individual class response rates as follows: CA 1: 78% (18 of 23), CA 2: 61% (14 of 23), and CA 3: 54% (12 of 22; Table 2). The time intervals between the simulation date and survey B for the CA 1, CA 2, and CA 3 classes were 4 months, 21 months, and 32 months, respectively. Residents responded that they had experienced real cases of hemodynamic instability due to major intraoperative blood loss requiring blood transfusions (94%, 40 of 43), that the simulation made them aware and prepared for such events (76%, 32 of 42), that they applied what they had learned in the simulation during real cases (70%, 30 of 43), and that the simulation experience improved patient care (77%, 33 of 43). There were no statistically significant differences between training classes for items 4–9 (Table 3) when analyzed for the percentage of respondents who agreed or the percentage who chose the correct answer.

**Table 2.**
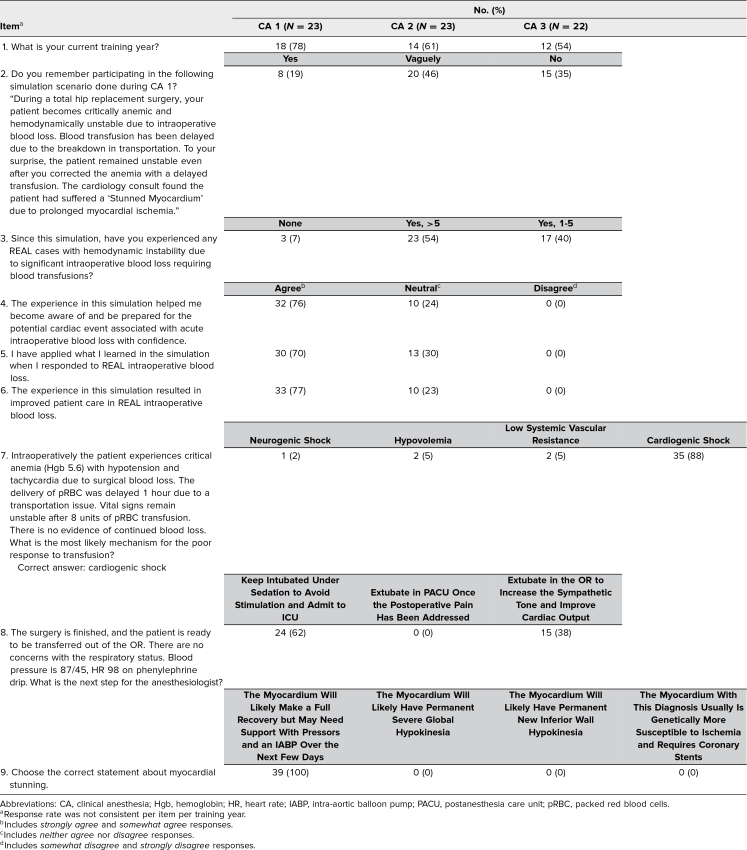
Distribution of Responses to Survey B (Follow-up Survey)

**Table 3.**
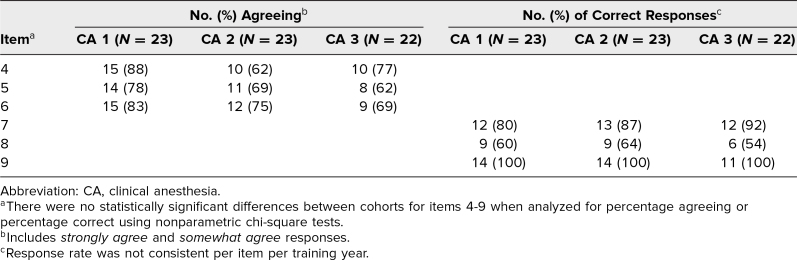
Distribution of Responses Based on Cohort Training Year

## Discussion

This simulation exercise allowed anesthesiology trainees to develop and practice skills regarding intraoperative cardiac complications and diagnosing and managing acute critical anemia in a psychologically safe simulated environment. The residents’ responses to survey A were positive regarding the simulation experience and relevance to their training (Kirkpatrick level 1). Survey B showed knowledge retention and increased self-confidence toward managing acute cardiac ischemia (Kirkpatrick level 2). It also revealed that residents applied this knowledge to real-world experiences of acute blood loss (Kirkpatrick level 3),^16^ which improved patient care. Knowledge was retained regardless of the time interval between the original simulation and survey B (Table 3). Although it cannot be concluded that this simulation alone taught residents how to address every acute myocardial ischemia associated with hemorrhage, we think exposure to such simulation scenarios early in training may increase awareness and preparedness for similar clinical situations.

Due to time constraints, the simulations were run in groups of three to five residents. Only one to two residents could be placed in the hot seat as the simulated anesthesiologist. The rest of the participants had to take on nonanesthesiologist roles. Although the residents’ uniformly positive responses indicated that they viewed the simulation as a valuable learning experience, the results were not stratified by those who were or were not in the hot seat. It is unclear if there was a difference in training effectiveness between residents in the hot seat and those playing nonanesthesiologist roles. Regardless of role within the simulation, all residents received the same debriefing experience. The importance of the role performed in the simulation is a future research topic and may be used to adjust future simulation setups.

Another limitation of this curriculum was the incomplete response rate. It has been shown that debriefings that occur immediately after simulations are more effective as the emotions and memories associated with the simulation can fade quickly,^17^ and so, a similar approach should be applied to survey A. As for survey B, collecting the survey results in person rather than online may increase the response rate.

There were no noted physical limitations with this curriculum that the participants emphasized. In the future, with the increased utilization of cardiac ultrasound in the OR setting, we plan to allow learners to order a transthoracic echocardiogram or transesophageal echocardiogram when they suspect a cardiac compromise (Appendix F).^18–21^ We plan to then bring in an echocardiogram simulator displaying a hypokinetic heart suggestive of stunned myocardium.^19,21^ A short video displaying similar ultrasound results can also be shown if such resources are unavailable. By adding these resources, we aim to simulate the most realistic OR environment possible with a full suite of supportive devices and services.

We chose not to grade or rank residents as this scenario was strictly a formative learning exercise. Thus, we do not have objective measures of performance to present, as the survey results are subjective data based on the learner's perception of efficacy. In survey A, a *t* test comparing items 1, 3, and 5 to items 2, 4, and 6 would not be appropriate as items 2, 4, and 6 assess not a specific level of clinical confidence but rather how much participants’ confidence improved because of the simulation. Given that *n* = 30 is the standard minimum for educational research, we are unlikely to gather a meaningful effect size calculation with one cohort (*n*s = 23 to 26), although we may combine cohorts over time.

We conclude that this simulation on acute blood loss and delay of blood products causing a stunned myocardium is of value to anesthesiology residents, can increase knowledge and retention of these topics, and offers knowledge that residents can apply to real-world cases going forward, thus meeting Kirkpatrick levels 1, 2, and 3 in evaluation training.

## Appendices


Stunned Myocardium Simulation Case.docxInfo for Patient.docxInfo for Anesthesiologist.docxInfo for Surgeon.docxIntraop POC Results.docxIntraop Cardiac US.docxCritical Actions Checklist.docxDebriefing Materials.docx
*All appendices are peer reviewed as integral parts of the Original Publication.*
