# Supplementary material for: Stunned Myocardium as a Sequela of Acute Severe Anemia: An Adult Simulation Case for Anesthesiology Residents
Source: MedEdPORTAL. 2024 Sep 6;20:11432. doi: 10.15766/mep_2374-8265.11432 (PMC11377552; doi:10.15766/mep_2374-8265.11432)
Supplement: Supplementary file 1 — Stunned Myocardium Simulation Case.docxInfo for Patient.docxInfo for Anesthesiologist.docxInfo for Surgeon.docxIntraop POC Results.docxIntraop Cardiac US.docxCritical Actions Checklist.docxDebriefing Materials.docx [file mep_2374-8265.11432-s001.zip › D. Info for Surgeon.docx]

**Appendix D**

***Your role is the Surgeon.*** *You have* ***5 minutes*** *to review the information with additional verbal explanations for any questions. You will be provided with a headset and occasionally instructed to help* ***redirect the scenario*** *or* ***provide additional information*** *by the facilitator.*

***Information for Surgeon***

73-year-old male presents to day surgery for elective **right total hip replacement surgery**.

He has a significant past medical history of coronary artery disease (CAD), hypertension (HTN), and chronic obstructive pulmonary disease (COPD). The patient’s functional capacity has been limited due to chronic hip pain, and he lives a primarily sedentary lifestyle.

1. *During the elective right total hip replacement surgery, your patient might have lost a fair amount of blood. When the anesthesiologist asks if you are losing blood, you attribute most of the fluid in the canister to irrigation.*
2. *You cannot halt the surgery because it is bleeding from the marrow, and the only way to stop the bleeding is to cement the instrument.*
3. *The blood transfusion has been ordered. You (Surgeon) and the anesthesiologist have agreed to start the case before the blood has arrived in the operating room because the patient’s starting hematocrit was normal (47%).*

**Allergies:** penicillin – hives and wheezing

**Past Medical History:**

- HTN
- COPD
- CAD

1. Anterior wall myocardial infarction 5 years ago, placed 2 coronary stents
2. Stable angina, most often with anxiety and always relieved with sublingual nitroglycerin
3. The patient sees a cardiologist regularly who states that the patient is optimally medically managed
4. Cardiac echo: Normal valve function, increased left atrial and left ventricular size,

hypokinetic anteroseptal wall, estimated ejection fraction = 47%

- Tobacco Use: 2 1/2 packs of cigarettes per day for 41 years
- Alcohol Use: Drinks 2-4 beers each night, denies history of delirium tremens
- Denies intravascular drug use

**Past Surgical / Anesthetic History:**

- Cardiac catheterization with 2 stents placement as above, no complications
- Transurethral resection of the prostate with no complications
- Tibial fracture - general anesthesia with no complications
- No family history of anesthetic problems

**Current Medications:**

He takes aspirin daily but stopped taking it 7 days ago as per the ortho clinic nurse’s instruction. He was also told to stop the rest of the meds this morning at the clinic.

- enalapril
- metoprolol
- aspirin
- furosemide
- nitroglycerin sublingual
