# Supplementary material for: Stunned Myocardium as a Sequela of Acute Severe Anemia: An Adult Simulation Case for Anesthesiology Residents
Source: MedEdPORTAL. 2024 Sep 6;20:11432. doi: 10.15766/mep_2374-8265.11432 (PMC11377552; doi:10.15766/mep_2374-8265.11432)
Supplement: Supplementary file 1 — Stunned Myocardium Simulation Case.docxInfo for Patient.docxInfo for Anesthesiologist.docxInfo for Surgeon.docxIntraop POC Results.docxIntraop Cardiac US.docxCritical Actions Checklist.docxDebriefing Materials.docx [file mep_2374-8265.11432-s001.zip › E. Intraop POC Results.docx]

**Appendix E**

*The ordering Anesthesiologist can receive the point-of-care blood test results within* ***1 minute***.

***Intraoperative Point-Of-Care Blood Test Results***

**Result 1**: To be provided to the Anesthesiologist upon request **after the significant loss of blood**.

pH 7.283

PaCO_2_ 36 mm Hg

PaO_2_ 416 mm Hg

HCO3 15 mEq/L

Base Excess (BE) - 4 mEq/L

Hematocrit (Hct) 18 %PCV

Hemoglobin (Hgb) 5.6 g/dL

Sodium (Na) 139 mEq/L

Potassium (K) 3.7 mEq/L

Ionized Calcium (iCa) 1.12 mmol/L

Glucose (Glu) 106 mg/dL

**Result 2**: To be provided to the Anesthesiologist upon request **after the blood transfusion**.

pH 7.326

PaCO_2_ 38 mm Hg

PaO_2_ 422 mm Hg

HCO3 20 mEq/L

Base Excess (BE) - 2 mEq/L

Hematocrit (Hct) 32 %PCV

Hemoglobin (Hgb) 10.6 g/dL

Sodium (Na) 141 mEq/L

Potassium (K) 3.9 mEq/L

Ionized Calcium (iCa) 1.03 mmol/L

Glucose (Glu) 99 mg/dL
