# Supplementary material for: Stunned Myocardium as a Sequela of Acute Severe Anemia: An Adult Simulation Case for Anesthesiology Residents
Source: MedEdPORTAL. 2024 Sep 6;20:11432. doi: 10.15766/mep_2374-8265.11432 (PMC11377552; doi:10.15766/mep_2374-8265.11432)
Supplement: Supplementary file 1 — Stunned Myocardium Simulation Case.docxInfo for Patient.docxInfo for Anesthesiologist.docxInfo for Surgeon.docxIntraop POC Results.docxIntraop Cardiac US.docxCritical Actions Checklist.docxDebriefing Materials.docx [file mep_2374-8265.11432-s001.zip › F. Intraop Cardiac US.docx]

**Appendix F**

***Intraoperative Cardiac Ultrasound Results***

Intraoperative cardiac ultrasound results are available for diagnostic aid upon the anesthesiologist’s request when they strongly suspect intraoperative cardiogenic shock due to prolonged anemia. The results can be presented in one of the following three methods depending on the equipment availability and the learner’s knowledge and skill level.

1. **Transthoracic Echocardiogram (TTE) or Transesophageal Echocardiogram (TEE) Simulators.** The study results can be programmed to display graphical results of Global Hypokinesis similar to the findings depicted in 3. The anesthesiologist should be competent in operating the TTE or TEE and interpret the results. This diagnostic activity will take approximately **5 minutes.**
2. **Video Clip.** A video clip displaying TTE or TEE results of Global Hypokinesis similar to the findings in 3 can be shown. The anesthesiologist has to interpret the results by viewing the video. This diagnostic activity will take approximately **2 minutes.**
3. **Descriptive Interpretations of the Ultrasound Results.** This information can be provided as a confirmation after the learner has accomplished one of the above two diagnostic activities. Alternatively, this information can be provided if the above two methods are unavailable during the simulation or if the learner anesthesiologist's skill level is not ready to operate TTE/TEE or interpret the ultrasound images. This diagnostic activity will take approximately **1 minute.**

**TTE Reading**: Increased left atrial and left ventricular size with concentric hypertrophy, right-sided chambers are normal in size and dimensions, global hypokinesia of myocardial wall, estimated EF = 18%.

**TEE Reading**: Increased left atrial and left ventricular size with concentric hypertrophy, right-sided chambers are normal in size and dimensions, global hypokinesia of myocardial wall, estimated EF = 18%. The left atrial appendage is clean without any clot or smoke effect. Mitral, tricuspid, and pulmonic valves are structurally normal. Mild aortic stenosis.
