# Supplementary material for: Stunned Myocardium as a Sequela of Acute Severe Anemia: An Adult Simulation Case for Anesthesiology Residents
Source: MedEdPORTAL. 2024 Sep 6;20:11432. doi: 10.15766/mep_2374-8265.11432 (PMC11377552; doi:10.15766/mep_2374-8265.11432)
Supplement: Supplementary file 1 — Stunned Myocardium Simulation Case.docxInfo for Patient.docxInfo for Anesthesiologist.docxInfo for Surgeon.docxIntraop POC Results.docxIntraop Cardiac US.docxCritical Actions Checklist.docxDebriefing Materials.docx [file mep_2374-8265.11432-s001.zip › G. Critical Actions Checklist.docx]

**Appendix G**

**Critical Actions Checklist**

**1. Initial Patient Evaluation**

- Preop the patient and collect pertinent information.
- Recognize the increased risk of ischemic heart events due to the patient’s pre-existing coronary artery disease and the implications of stopping aspirin and other antihypertensive medicines before the surgery.

**2. Intraoperative Crisis Management**

- Recognize the signs and symptoms of acute surgical blood loss.
- Communicate with the surgeons to estimate the amount of current and future blood loss.
- Perform physical exams (e.g., palpebral conjunctiva, nail bed) to assess the severity of anemia.
- Order point-of-care labs to assess the severity of anemia.
- Order pRBCs.
- Order unmatched pRBCs once recognizing that the treatment of anemia cannot be delayed.
- Differentiate between hypovolemia vs. anemia vs. cardiogenic shock.
- Treat cardiogenic shock without pRBC or IABP (crystalloid, colloid, vasoconstrictors, inotropes).

**3. Post-operative Management**

- Order a bedside TTE or TEE.
- Make the decision to keep the patient intubated due to hemodynamic instability.
- Consult cardiology.
