# Supplementary material for: Stunned Myocardium as a Sequela of Acute Severe Anemia: An Adult Simulation Case for Anesthesiology Residents
Source: MedEdPORTAL. 2024 Sep 6;20:11432. doi: 10.15766/mep_2374-8265.11432 (PMC11377552; doi:10.15766/mep_2374-8265.11432)
Supplement: Supplementary file 1 — Stunned Myocardium Simulation Case.docxInfo for Patient.docxInfo for Anesthesiologist.docxInfo for Surgeon.docxIntraop POC Results.docxIntraop Cardiac US.docxCritical Actions Checklist.docxDebriefing Materials.docx [file mep_2374-8265.11432-s001.zip › H. Debriefing Materials.docx]

**Appendix H**

**Debriefing Materials**

**General Questions:**

1. What do you think went well?
2. What did you have difficulty with?
3. Is there anything you would change?

**Specific Clinical Questions Pertinent to this Simulation:**

1. What are the perioperative implications for ischemic heart disease and coronary stents?
2. What are the implications of stopping the daily aspirin or antihypertensive medicine before surgery?
3. How should we communicate with the surgical team when the patient is progressively declining? Share your experiences in communicating assertively.
4. How do you differentiate unstable hemodynamics caused by hypovolemia vs. anemia vs. cardiogenic shock?
5. How do you manage acute intraoperative blood loss, and what strategies can be implemented if pRBCs are not immediately available?
6. How do you manage cardiogenic shock? What is the best treatment to maintain perfusion to critical organs without further stressing the heart that has already been ischemic?
7. Why should we keep this patient intubated? What will happen if we extubate?
8. What is myocardial stunning, and what is its clinical relevance?

The following elements and talking points are recommended during the debriefing with the learners.

1. **Evaluation of the patient in the preoperative area:** This scenario allows the learner to demonstrate preparedness for complications while developing their anesthetic plan. The plan should specify the need for additional lines, monitors, and type and screen/type and cross of the blood product. Discuss the implications of stopping the daily aspirin or antihypertensive medicine preoperatively. Discuss if the anesthesiologist missed any information regarding the patient’s history or anesthetic plan.
2. **Differentiating hypovolemia vs. anemia vs. cardiogenic shock:** The learner anesthesiologist should discuss the differences in the signs and symptoms of hypovolemia, anemia, and cardiogenic shock. These three conditions all present with hypotension and tachycardia. The learners should discuss what diagnostic measures could be helpful. Physical exams, such as checking the palpebral conjunctiva and nail beds to assess anemia or feeling the pulse if they believe the blood pressure reading is not reliable, are something they can quickly perform without any instruments. They can also discuss how these conditions differ in response to fluid challenge and vasoactive pressors. The significance of utilizing point-of-care blood tests and point-of-care ultrasound (POCUS) should be addressed.
3. **Adaptation to the delay in receiving blood products:** The learner should discuss how to respond to unexpected intraoperative obstacles, such as learning that type and cross or type and screen have not been ordered or the blood not arriving on time while experiencing critical anemia. Confirm own institution’s policy about ordering uncrossmatched blood for emergency transfusion. The learner should share their thought process in devising an alternative plan.
4. **Management of cardiogenic shock under critical anemia complicated with delay in blood transfusion:** The learners should discuss the intraoperative cardiogenic shock treatment strategy. What is the best treatment to maintain perfusion to critical organs without putting further stress on the heart that has already been ischemic? What determines candidacy for extubating versus keeping intubated postoperatively? Learners should also discuss that postoperative care of cardiogenic shock patients may include mechanical support systems (MCS) such as an intra-aortic balloon pump, percutaneous LV MCS, or extracorporeal membrane oxygenation (ECMO). Learners should recognize that in the acute setting of our scenario, these resources are not viable immediate treatments in the operating room for cardiac shock, which is why they were excluded from the simulation.
5. **Communication with the surgical team:** The learners should discuss communicating effectively with the surgical team, particularly when the patient is progressively declining. Participants may share their experiences in communicating assertively and navigating challenging personalities.
