# Supplementary material for: Stunned Myocardium as a Sequela of Acute Severe Anemia: An Adult Simulation Case for Anesthesiology Residents
Source: MedEdPORTAL. 2024 Sep 6;20:11432. doi: 10.15766/mep_2374-8265.11432 (PMC11377552; doi:10.15766/mep_2374-8265.11432)
Supplement: Supplementary file 1 — Stunned Myocardium Simulation Case.docxInfo for Patient.docxInfo for Anesthesiologist.docxInfo for Surgeon.docxIntraop POC Results.docxIntraop Cardiac US.docxCritical Actions Checklist.docxDebriefing Materials.docx [file mep_2374-8265.11432-s001.zip › B. Info for Patient.docx]

**Appendix B**

***Your role is the patient.*** *Review the following information first. You are receiving an elective right total hip replacement surgery today. You will meet with your anesthesiologist(s) in the pre-operative area and discuss what type of anesthesia you will be receiving for this surgery. You have* ***2 minutes*** *to review this information sheet and* ***5 minutes*** *to accomplish the pre-operative encounter. You will then be assigned a different role and be moved to the Operating Room.*

***Information for Patient***

73-year-old male presents to day surgery for elective **right total hip replacement surgery**.

He has a significant past medical history of coronary artery disease (CAD), hypertension (HTN), and chronic obstructive pulmonary disease (COPD). The patient’s functional capacity has been limited due to chronic hip pain, and he lives a primarily sedentary lifestyle.

**Allergies:** penicillin – hives and wheezing

**Past Medical History:**

- HTN
- COPD
- CAD

1. Anterior wall myocardial infarction 5 years ago, placed 2 coronary stents
2. Stable angina, most often with anxiety and always relieved with sublingual nitroglycerin
3. The patient sees a cardiologist regularly who states that the patient is optimally medically managed
4. Cardiac echo: Normal valve function, increased left atrial and left ventricular size,

hypokinetic anteroseptal wall, estimated ejection fraction = 47%

- Tobacco Use: 2 1/2 packs of cigarettes per day for 41 years
- Alcohol Use: Drinks 2-4 beers each night, denies history of delirium tremens
- Denies intravascular drug use

**Past Surgical / Anesthetic History:**

- Cardiac catheterization with 2 stents placement as above, no complications
- Transurethral resection of the prostate with no complications
- Tibial fracture - general anesthesia with no complications
- No family history of anesthetic problems

**Current Medications:**

He takes aspirin daily but stopped taking it 7 days ago as per the ortho clinic nurse’s instruction. He was also told to stop the rest of the meds this morning at the clinic.

- enalapril
- metoprolol
- aspirin
- furosemide
- nitroglycerin sublingual

**Physical Examination:**

General: Healthy adult

Weight, Height: 98 kg, 182 cm

Vital Signs: HR 76, BP 180/97, RR 14, Sp0_2_ 94%

Airway: Upper & lower dentures, full cervical range of motion, 3-fb oral opening, 3-fb mandible, Mallampati Class 2

Lungs: Distant and equal bilateral breath sounds

Heart: Regular rate and rhythm. S1 and S2 are of normal intensity. No S3, S4, murmur, or rub

**Laboratory:**

Chest X-ray: There are no infiltrates. The aortic arch is calcified, and the heart border is slightly

enlarged. The pulmonary artery is prominent.

12-Lead Electrocardiogram: Normal sinus rhythm, ST changes consistent with old MI, age undetermined

Hematocrit: 47%

Electrolytes: WNL
