# Supplementary material for: Stunned Myocardium as a Sequela of Acute Severe Anemia: An Adult Simulation Case for Anesthesiology Residents
Source: MedEdPORTAL. 2024 Sep 6;20:11432. doi: 10.15766/mep_2374-8265.11432 (PMC11377552; doi:10.15766/mep_2374-8265.11432)
Supplement: Supplementary file 1 — Stunned Myocardium Simulation Case.docxInfo for Patient.docxInfo for Anesthesiologist.docxInfo for Surgeon.docxIntraop POC Results.docxIntraop Cardiac US.docxCritical Actions Checklist.docxDebriefing Materials.docx [file mep_2374-8265.11432-s001.zip › A. Stunned Myocardium Simulation Case.docx]

| **Appendix A**  **SIMULATION CASE TITLE: Stunned Myocardium as a Sequela of Acute Severe Anemia: An Adult Simulation Case for Anesthesiology Residents**    AUTHORS: David Ryusuke Okano, MD, PhD^1*^, Bryan Ko^2^, Marelle Giuliano, MD^3^, Sally Mitchell EdD, MMSc^4^, Johnny Cartwright^5^, Christopher Moore^6^ and Tanna Boyer, DO, MS^4^  ^1^ Assistant Professor, Department of Anesthesia, Tokyo Women’s Medical University  ^2^ Fourth Year Medical Student, Indiana University School of Medicine  ^3^ Resident Physician, Department of Anesthesia, Indiana University School of Medicine  ^4^ Associate Professor, Department of Anesthesia, Indiana University School of Medicine  ^5^ Associate Director of Simulation, Department of Anesthesia, Indiana University School of Medicine  ^6^ Simulation Technician, Department of Anesthesia, Indiana University School of Medicine  * Correspondence: [okano.ryusuke@twmu.ac.jp](mailto:okano.ryusuke@twmu.ac.jp) | |
| --- | --- |
| **PATIENT: E.F. 73-year-old male**  **CHIEF COMPLAINT: Elective Right Total Hip Replacement Surgery**  **PHYSICAL SETTING: Preoperative Unit and Operating Room** | |
|  | |
| **Brief Narrative Description of Case** | The first scene begins with a patient presenting for elective total hip replacement surgery. The learner anesthesiologist is to perform a pre-operative evaluation and learn that the patient, a 73-year-old male, has a history of CAD, MI, coronary stents x 2, HTN, and COPD. He has ongoing stable angina, controlled by sublingual NTG as needed. He is non-compliant with his daily aspirin. He stopped all other daily medications this morning (enalapril, metoprolol, furosemide). He is sedentary, as activity is limited by chronic hip pain. A pre-operative cardiac ultrasound study demonstrates increased LA and LV size, hypokinetic anteroseptal wall, and EF=47%. Pre-operative CXR with calcified aortic arch, enlarged heart border, and prominent pulmonary artery. ECG with ST changes consistent with old MI. His past medical history is otherwise significant for tobacco use (2 ½ PPD for 41 years) and daily alcohol use (2-4 beers a night). He has had previous surgeries, including TURP and tibial fracture repair, with no associated surgical or anesthetic complications.  Following the pre-operative evaluation, the second scene begins in the OR with general anesthesia induction. It was decided to start the case without the blood ready in the OR because the patient’s starting hematocrit was 47%. Induction goes smoothly with no complications. As the surgery continues, the suction canister quickly fills up with blood. The learner anesthesiologist is to address the concern for blood loss with the surgical team, who explains that the volume could be attributed to irrigation. The learner anesthesiologist recognizes the need for pRBC soon; however, the ordered pRBC has not yet been delivered to the OR due to an unknown issue. The patient’s vitals become unstable, and the learner anesthesiologist communicates with the surgical team accordingly. After asking if the surgery can be stopped until the learner anesthesiologist can stabilize the vital signs, the surgical team states that they must continue, as blood is actively emanating from the bone marrow and needs to be cemented with the orthopedic instrument. Concerned for critical anemia, the learner anesthesiologist obtains a POC hemoglobin and discovers the patient’s hematocrit is 18 (hemoglobin 5.6).  The surgery ends, but the patient’s hemodynamic stability continues to decline, with associated ischemic changes and frequent PVCs noted on the ECG. Noticing these changes, the learner anesthesiologist orders unmatched O-negative blood stat. The circulator then explains that the blood transportation system is down and there will be an unknown delay in the delivery of all blood products. The learner anesthesiologist then must manage the acutely anemic patient without blood products. Vitals demonstrate hypotension (BP 50/32 to 82/45) and tachycardia (HR 110-128).  After a long wait, the blood products finally arrives and blood was administered in the hopes of achieving hemodynamic stability; however, the patient’s vitals remain unstable (BP 81/45, HR 118, ECG with frequent PVCs).  At this point, the learner anesthesiologist suspects cardiogenic shock associated with a cardiac ischemic event. Cardiac ultrasound (TEE or TTE) reveals global hypokinesis of the heart. The learner anesthesiologist decides to keep the patient intubated and admit to the ICU. A cardiology consult confirms the diagnosis of stunned myocardium due to prolonged myocardial ischemia because of the delay in treating critical anemia. The patient is supported with an aortic balloon pump for approximately 5 days and recovers well. He is discharged with no irreversible myocardial damage. |
| **Primary Learning Objectives** | 1. Perform an efficient patient encounter in the preoperative area, focusing on the implications of coronary artery disease (CAD) and antiplatelet therapy for coronary stents. 2. Establish an anesthesia plan appropriate for the proposed surgery and the presented patient. 3. Recognize the signs and symptoms of acute surgical blood loss and treat accordingly. 4. Demonstrate effective communication with surgeons and the surgical team in the operating room. 5. Adapt to an unexpected delay in blood product delivery and manage unstable hemodynamics. 6. Differentiate between hypovolemic shock vs. anemic shock vs. cardiogenic shock. 7. Manage intraoperative cardiogenic shock and subsequent ischemic cardiac events due to untreated anemia. 8. Demonstrate effective communication with the cardiologist. |
| **Critical Actions** | 1. Initial Patient Evaluation and Induction of General Anesthesia   - Preop the patient and collect pertinent information. - Recognize the increased risk of ischemic heart events due to the patient’s pre-existing coronary heart disease and the nature of the surgery and prepare with proper intravenous lines and an arterial line monitor.   2. Intra-operative Crisis Management   - Recognize the signs and symptoms of acute surgical blood loss, including the accumulation of blood in the suction canister and deteriorating vital signs. - Communicate with the surgeons to estimate the amount of current and future blood loss. - Order an ABG and H/H to assess the severity of anemia. - Treat unstable hemodynamics due to anemia. - Order red blood cell products. - Recognize the new onset of an ischemic cardiac event. - Recognize that the treatment of anemia cannot be delayed and order unmatched red blood cell products. - Devise the best plan to treat the cardiogenic shock while waiting for the delayed blood products. - Transfuse blood once the blood product arrives.   3. Post-operative Management   - Order a bedside TTE or TEE. - Make the decision to leave the patient intubated due to unstable hemodynamics. - Consult cardiology. |
| **Learner Preparation** | Learners are expected to have prior experience in operating room anesthesia and have rotated in the intensive care unit setting (usually in intern/PGY1 year). |

| Initial Presentation | |
| --- | --- |
| **Initial vital signs**  *These are the vital signs during the pre-operative assessment.* | HR 76, BP 180/97, SpO2 94%, RR 14 |
| **Overall Appearance**  *What the learners see when they first enter each scene.* | First Scene (Pre-operative Area): The patient is waiting as the learner enters the room, dressed in a hospital gown. |
|  | Second Scene (Operating Room): The patient mannequin is supine on the OR table, awake, and awaiting induction. The patient is pre-draped with a fenestration over the right thigh. A surgeon and other OR staff are in the room. |
| Participants and their Simulation roles | Participant #1: Anesthesiologist (Referred to as “Learner” in the Instructor Notes)  Participant #2: Patient* →Surgeon B  Participant #3: Surgeon A  Participant #4: Circulating Nurse**  Participant #5: Scrub Tech**  In addition to Participants #1- #5, the facilitator runs the simulation, and the simulation technician controls the simulator equipment. The facilitator and simulation technician stay out of sight throughout the entire scenario.  *After the first scene (Pre-operative encounter), the Patient switches to Surgeon B.  **Extra simulation faculties can play these roles if the total number of Participants is three or less. |
| **HPI**  *Information provided by the facilitator* | 73-year-old male presents to day surgery for elective right total hip replacement surgery. He has a significant past medical history of coronary artery disease (CAD), hypertension (HTN), and chronic obstructive pulmonary disease (COPD). The patient’s functional capacity has been limited due to chronic hip pain, and he lives a primarily sedentary lifestyle.    Allergies: penicillin – hives and wheezing  Past Medical History:   - HTN - COPD - CAD  1. Anterior wall myocardial infarction 5 years ago, placed 2 coronary stents 2. Stable angina, most often with anxiety and always relieved with sublingual nitroglycerin 3. The patient sees a cardiologist regularly who states that the patient is optimally medically managed 4. Cardiac echo: Normal valve function, increased left atrial and left ventricular size, hypokinetic anteroseptal wall, estimated ejection fraction = 47%  - Tobacco Use: 2 and a half packs of cigarettes per day for 41 years - Alcohol Use: Drinks 2-4 beers each night, denies history of delirium tremens - Denies intravascular drug use     Past Surgical / Anesthetic History:   - Cardiac catheterization with 2 stents placement as above, no complications - Transurethral resection of the prostate with no complications - Tibial fracture - general anesthesia with no complications - No family history of anesthetic problems     Current Home Medications:  He takes aspirin daily but stopped taking it 7 days ago as per the orthopedics clinic nurse’s instruction. He was also told to stop the rest of the meds this morning at the clinic.   - enalapril - metoprolol - aspirin - furosemide - nitroglycerin sublingual |

| **Physical Examination** | |
| --- | --- |
| **General** | Alert, awake, resting in a patient bed |
| **HEENT** | PERRL, upper and lower dentures, 3 fb oral opening |
| **Neck** | Supple, FROM, 3 fb mandible |
| **Lungs** | Distant but equal breath sounds bilaterally |
| **Cardiovascular** | NSR, no murmurs, ECG with ST segment changes consistent with old MI, age undetermined |
| **Abdomen** | Soft, non-distended |
| **Neurological** | Alert and oriented x 3, no focal deficits |
| **Skin** | No erythema, bruising, or rashes |
| **GU** | N/A |
| **Psychiatric** | Cooperative, appropriate affect |
| Laboratory | |
| **Chest X-ray** | There are no infiltrates. The aortic arch is calcified, and the heart border is slightly  enlarged. The pulmonary artery is prominent. |
| **12-Lead Electrocardiogram** | Normal sinus rhythm, ST changes consistent with old MI, age undetermined |
| **Hematocrit** | 47% |
| **Electrolytes** | WNL |

| Instructor Notes - Changes and CASE Branch Points | | |
| --- | --- | --- |
| **Intervention / Time point** | **Change in Case** | **Additional Information** |
| **First Scene (Pre-operative Area):** The learner has 2 minutes to read through the patient information and the scheduled procedure (Appendix C). The learner then enters the pre-operative area to assess and consent the patient for 5 minutes. | The learner realizes the patient has a significant cardiac history. | The learner should understand the implications of the patient’s history of ischemic heart disease, coronary stents, and hypertension.  Upon completing the assessment and obtaining anesthesia consent, the learner will be instructed to move to the operating room.  The patient will be instructed to change the role to a surgeon and then move to the operating room. |
| **Second Scene (Operating Room):**  The learner induces the anesthesia. | Induction of the anesthesia starts. | The induction of anesthesia is smooth without complications. |
| 2 minutes after the induction (the learner is notified that 30 minutes have elapsed in the procedure): In the middle of the surgery, the learner notes the suction canister is quickly filling up with blood. The amount of blood appears concerning. The learner communicates with the surgical team and asks about blood loss. | The patient’s blood pressure starts to trend down mildly. The surgeons do not believe they have had such significant blood loss. It is unclear what volume is attributed to irrigation versus true blood loss. | If the learner does not note the canister filling up with blood, the facilitator will instruct the surgeons over the headsets to say, “Why is that canister so full?” to direct the learner’s attention to the canister. The learner should consider proactively securing larger-bore IV access, an arterial line. When the learner tries to confirm when the blood will be delivered to the OR, the circulating nurse replies, “I am not sure why it’s taking so long, but it should be arriving any moment.” |
| 1 minute later (the learner is notified that another 10 minutes have elapsed in the procedure): The patient’s vitals become unstable. The learner again communicates with the surgeons to see if the procedure can be held until the vitals are stabilized; however, the surgeons explain that they must continue since the active bleeding from the bone marrow does not stop until the surgical implant gets cemented in. | The patient develops significant hypotension and tachycardia. | The learner must start treating the hypotension by administering fluids and pressors. Due to previous concern for blood loss, critical anemia should be at the top of the differential. This should be confirmed by a point-of-care (POC) blood test. |
| The learner obtains a POC blood test result and discovers the patient’s hematocrit is 18 (hemoglobin 5.6). The learner asks for blood but was told it is still unavailable. | The patient’s vitals remains unstable and are consistent with hypovolemia and critical anemia due to acute blood loss. | Appendix E (POC blood test Result 1) to be shown to the learner. The learner must recognize that the patient needs a blood transfusion as soon as possible. If not already established, the learner should place an arterial line and add large-bore IV access. Consider further interventions with colloids, adjusting the anesthetic depth, and medications to manage the patient’s unstable vitals. |
| The surgeons finish the surgical procedure. There should be no additional blood loss from here on. | The patient continues to deteriorate with ischemic ECG changes and frequent PVCs. | Vitals remain unstable (BP 50/32mmHg to 82/45 mmHg, HR 110-128bpm). If the learner does not notice the ischemic changes on the ECG, the facilitator will instruct the surgeons to draw the learner’s attention to the ECG monitor. The learner must maintain perfusion of critical organs while trying to protect the heart as much as possible. The learner must realize the emergency need to order unmatched O-negative blood. |
| The learner orders unmatched O-negative blood stat. | It is discovered that the blood transportation system is down, and there will be an unknown delay in the delivery of all blood products. |  |
| After 2 minutes (the learner is notified that 50 minutes have elapsed after ordering the O-negative blood), the unmatched O-negative blood arrives to the OR. The learner rapidly transfuses several units of blood and re-checks the POC blood test results. | The POC ABG results show that the patient is no longer anemic. Despite blood transfusion, the patient’s hemodynamics show marginal improvement and remain unstable. | Appendix E (POC blood test Result 2, hemoglobin 10.6) to be shown to the learner. The patient remains hypotensive and tachycardic (BP 81/45, HR 118) with frequent PVCs. At this point, the learner must realize that the patient is in cardiogenic shock. If the learner struggles to make the correct diagnosis, the facilitator can make the surgeons ask questions such as “Why is the patient still unstable although the patient is no longer anemic?” “What can you do to find out the reason for this hypotension?” “How about cardiac ultrasound?” |
| The learner calls for echocardiography in the OR. | TTE/TEE results show global hypokinesia, which is the reason for cardiogenic shock. | TTE/TEE simulator, a video clip, or a written echocardiography result will be presented to the learner (Appendix F). |
| With the confirmed diagnosis of cardiogenic shock, the learner makes the decision not to extubate and admit the patient to the ICU. The cardiology team is also consulted. | The patient’s vital signs remain hypotensive and tachycardic but not worsening. | The vital signs will quickly deteriorate if the learner attempts to wake up and extubate the patient. The facilitator can make the surgeons to speak to the learner to rethink that decision.  The learner needs to provide a summary of the event to the cardiac intensivist. The facilitator can play the role of the cardiac intensivist over the phone. |
| The patient is admitted to the ICU. The cardiology team confirms that the patient has suffered from stunned myocardium due to prolonged anemia and ischemia. | An aortic balloon pump was placed for 5 days. The patient recovered and was discharged with no irreversible myocardial damage. | Learners should understand that prolonged critical anemia can cause reversible yet significant global hypokinesia, leading to cardiogenic shock that warrants timely diagnosis and intervention. |

Ideal Scenario Flow

Before starting the simulation, the learner (participant #1, anesthesiologist) is given time to familiarize themselves with the simulated OR environment. The patient (participant #2) and surgeon (participant #3) are briefed on their respective roles with Appendices B and D. The learner (participant #1) is then provided with Appendix C and instructed to perform a pre-operative evaluation and obtain anesthesia consent in the preoperative area (First Scene). The simulation will then transition to the operating room (Second Scene).

Upon entering the operating room, the patient lies on the operating table, awaiting induction. The learner knows the typed & crossed blood has not been delivered to the OR but agreed to start the case since the initial hematocrit was 47%. The patient has an 18G peripheral IV in the left upper extremity, and other appropriate ASA monitors are in place. Vitals are normal. Induction goes smoothly, and the airway is secured without complications.

As the surgery continues, the suction canister quickly fills with blood. The learner promptly notices this and begins communicating with the surgeon(s), who explains that the volume in the canister may be primarily due to irrigation versus actual blood loss. Soon after that, the patient’s vitals become unstable. The learner recognizes the deterioration and again communicates with the surgeon(s) to see if the surgery can be held until the learner can stabilize the patient. The surgeon(s) explain they must continue as there is active bleeding from the bone marrow, and they must continue to work to cement the orthopedic implant to stop bleeding. The learner becomes concerned about critical anemia and performs a POC blood test while starting to administer pressors and fluids. Critical anemia has been confirmed, with hematocrit being 18 (hemoglobin 5.6). The learner secures large-bore IV access and places an arterial line to prepare for further treatment.

The surgery has ended, but the blood has still not arrived for the patient. The patient’s vitals continue to decline (BP 50/32mmHg to 82/45mmHg, HR 110-128bpm) and begin to demonstrate signs of progressive hemodynamic instability with ischemic ECG changes and frequent PVCs. The learner strategizes the methods for maintaining the perfusion of critical organs while protecting the heart as much as possible. The learner proceeds to request an unmatched O-negative blood stat. The learner is then told that the blood transportation system is down and there is an unknown delay in the delivery of all blood products.

After a long wait, the unmatched O-negative blood finally arrives and is administered to the patient in hopes of achieving hemodynamic stability. Despite the anemia having been corrected after the blood transfusion, the patient’s vitals remain unstable (BP 81/45mmHg, HR 118bpm, ECG with frequent PVCs). At this point, the learner strongly suspects the patient is in cardiogenic shock and orders a cardiac ultrasound in the OR. The ultrasound result shows global hypokinesia, which is consistent with the clinical diagnosis of cardiogenic shock. The learner decides to keep the patient intubated and admit to the ICU due to hemodynamic instability.

The cardiology team evaluates the patient and confirms the diagnosis of stunned myocardium due to myocardial ischemia in the setting of prolonged critical anemia. An aortic balloon pump was placed for 5 days, and the patient recovered with no new irreversible myocardial damage. The simulation exercise is then completed, and the participants are appropriately debriefed.

Anticipated Management Mistakes

1. *Failure to recognize the acute blood loss and start prompt fluid resuscitation, and not efficiently communicating with the surgical team.* The learner may fail to notice the blood loss or attribute the volume to irrigation. They may fail to assertively express their concerns to the surgical team. The learner may also fail to obtain additional large-bore IV access and promptly initiate fluid resuscitation. Learners should make sure they perform other necessary diagnostic actions or interventions, such as arterial line placement or POC blood tests.
2. *Failure to make a quick decision to order unmatched red cells once recognizing the patient is becoming hemodynamically unstable due to cardiac ischemia.* The learner may struggle to realize that the patient is experiencing an underlying ischemic cardiac event and may underestimate the situation's urgency. They may continue to wait for crossmatched blood instead of ordering unmatched blood products, which might arrive sooner. Learners should always consider alternative plans and anticipate unexpected obstacles.
3. *Failure to recognize and treat the cardiogenic shock properly. (Such as administering beta blockers to treat tachycardia or giving excessive vasoconstrictors).* The learner may fail to realize that the patient’s deterioration and vital signs are consistent with cardiogenic shock. They may fail to initiate the appropriate medications or mistakenly administer medications exacerbating the patient’s condition. The learner should understand the proper management of cardiogenic shock.
4. *Waking up and extubating while the patient is still hemodynamically unstable.* The learner may not realize the detrimental impact of increased sympathetic activity and postoperative pain on the ischemic heart. Learners should understand the indications for leaving a patient intubated and admitting them to the ICU.
